# Construction of Recombinant *Escherichia coli* Expressing Ammonia Assimilation Genes and Evaluation of Its Effect on Removing Ammonium Nitrogen (NH_4_^+^-N)

**DOI:** 10.3390/microorganisms13122646

**Published:** 2025-11-21

**Authors:** Pan Pan, Yongkun Yang, Runxuan Shi, Yulin Kang, Hanli Xu, Xiyu Cheng, Qiong Yan, Honggang Hu

**Affiliations:** 1 College of Life Sciences and Bioengineering, School of Physical Science and Engineering, Beijing Jiaotong University, Beijing 100044, China; 20121611@bjtu.edu.cn (P.P.); 23121766@bjtu.edu.cn (Y.Y.); 21121603@bjtu.edu.cn (R.S.); xuhanli@bjtu.edu.cn (H.X.); xycheng@bjtu.edu.cn (X.C.); 2 College of Biotechnology, Tianjin University of Science and Technology, Tianjin 300457, China; 3 Chinese Research Academy of Environmental Sciences, Beijing 100012, China; kangyulin@craes.org.cn

**Keywords:** ammonia assimilation, *Escherichia coli*, gene co-expression, removing ammonium nitrogen

## Abstract

Treating wastewater with high ammonium nitrogen (NH_4_^+^-N) is a public and environmental priority. Unlike nitrification–denitrification, ammonia assimilation channels NH_4_^+^-N into the glutamate biosynthetic pathway, avoiding gaseous nitrogen species (NOx, N_2_). Here we engineered *Escherichia coli* to enhance ammonia assimilation by co-expressing three key genes, *gdhA*, *glnA*, and *guaA*. The genes were synthesized, cloned into expression plasmids, and introduced into *E. coli* BL21 for IPTG-inducible expression. Expression of the target proteins at expected sizes was observed, and NH_4_^+^-N removal was assessed in flask fermentations. Recombinant strains exhibited significantly higher NH_4_^+^-N reduction than the empty vector control; among them, BL21(pET-*gdhA*-*glnA*-*guaA*) performed best, achieving a maximum removal efficiency of 90.09% under the tested conditions. These results indicate that reinforcing the glutamate pathway through multi-gene co-expression can effectively lower NH_4_^+^-N in culture and provide a basis for developing recombinant bacteria for practical sewage treatment.

## 1. Introduction

Human activities have introduced substantial quantities of nitrogen into aquatic environments, leading to serious ecological impacts [1]. Developing reliable and efficient nitrogen removal technologies is therefore essential for protecting water quality. Among existing approaches, biological nitrogen removal is widely applied due to its relatively low cost and operational simplicity [2,3,4]. Conventional systems depend on nitrifying and denitrifying microorganisms to sequentially oxidize ammonia to nitrate and subsequently reduce it to N_2_. However, these processes typically require controlled environmental conditions and separate operational stages, which increase both complexity and cost [5,6]. Moreover, autotrophic nitrifiers often display slow growth rates in environments with high organic loads, further limiting treatment efficiency [7,8,9,10].

Simultaneous heterotrophic nitrification–aerobic denitrification (HN-AD) offers a promising alternative, enabling concurrent removal of ammonia, nitrate, and organic carbon within a single reactor. HNAD strains generally grow rapidly, tolerate higher organic content, and can integrate multiple nitrogen transformation pathways [11,12,13]. In some of these strains, the dissimilatory nitrate reduction to ammonium (DNRA) pathway has also been reported, which contributes to nitrogen removal through the incorporation of NH_4_^+^ into biomass [14,15].

Ammonia assimilation plays a central role in this process, with NH_4_^+^ incorporated into cellular components via specific metabolic pathways [16,17,18]. It can also influence nitrate metabolism through assimilatory nitrate reduction and by modulating nitrification [19,20,21]. Microorganisms typically assimilate ammonia via two main routes: the glutamate dehydrogenase (GDH) pathway and the glutamine synthetase (GS)–glutamate synthase (GOGAT) pathway [16,18,22,23,24,25]. The *gdhA* gene encodes GDH, an enzyme ubiquitous in animals, plants, and microbes, which catalyzes the reversible oxidative deamination of L-glutamate to 2-oxoglutarate and ammonia [22,23,24].

Glutamine synthetase enzymes are categorized into GSI, GSII, and GSIII types based on sequence length, with the GSI type (~55 kDa per subunit, encoded by *glnA*) found in many bacteria and other prokaryotes [25,26,27]. Recent studies have identified fine-tuned regulation of GS activity and assembly, including 2-oxoglutarate–dependent activation [28]. Cellular ammonium levels are also regulated by Amt/Mep/Rh family transporters, whose functions vary across organisms [18,29,30]. The *glnA* gene, encoding GSI, has been positively linked to nitrogen removal efficiency in both engineered and natural systems, responding to factors such as the carbon/nitrogen ratio (COD/N) and other operational parameters [31,32]. Genome analyses have shown that certain HN-AD bacteria, such as *Klebsiella* sp. TSH15, harbor a complete ammonia assimilation module, including *amt*, *glnA/H/Q*, *gdhA*, and *gltB/D*, enabling conversion of NH_4_^+^-N to glutamate via the glutamate pathway [14,33]. Multi-omics studies further confirm the interconnection of these genes within simultaneous nitrogen removal systems.

In the present work, we applied genetic engineering to develop *E. coli* strains capable of efficient ammonium removal. These engineered strains were designed for rapid growth, ease of cultivation, adaptability to sewage environments, and enhanced ammonium degradation capacity. Expression conditions were optimized, and their NH_4_^+^-N removal performance in simulated wastewater was evaluated. Specifically, we targeted two primary ammonium-assimilation nodes—glutamate dehydrogenase (GDH; *gdhA*) and glutamine synthetase (GS; *glnA*)—and also included *guaA* (GMP synthetase), which not act as an assimilation enzyme but as a downstream nitrogen sink that consumes glutamine in nucleotide biosynthesis. This design aims to reinforce assimilation while potentially channeling assimilated nitrogen into growth-associated metabolism.

High-strength ammonium under low C/N and inhibitory co-contaminants (e.g., antibiotics, free ammonia, and free nitrous acid) constrains conventional nitrification–denitrification and even HN-AD, due to oxygen and carbon demand as well as toxicity management. In such scenarios, ammonium assimilation functions as a practical nitrogen sink by channeling NH_4_^+^ into biomass. However, most prior engineering efforts strengthened single nodes (either GDH or the GS–GOGAT cycle), leaving open whether parallel reinforcement of GDH and GS within one chassis, together with a downstream glutamine-consuming sink in nucleotide biosynthesis (e.g., *guaA*), can broaden the workable ammonium window and improve robustness under dynamic influent. Notably, dissimilatory nitrate reduction to ammonium (DNRA) does not remove by itself total nitrogen; net nitrogen removal arises when NH_4_^+^ is assimilated into biomass or otherwise sequestered, which motivates the assimilation-oriented engineering pursued here.

**Figure 1:**
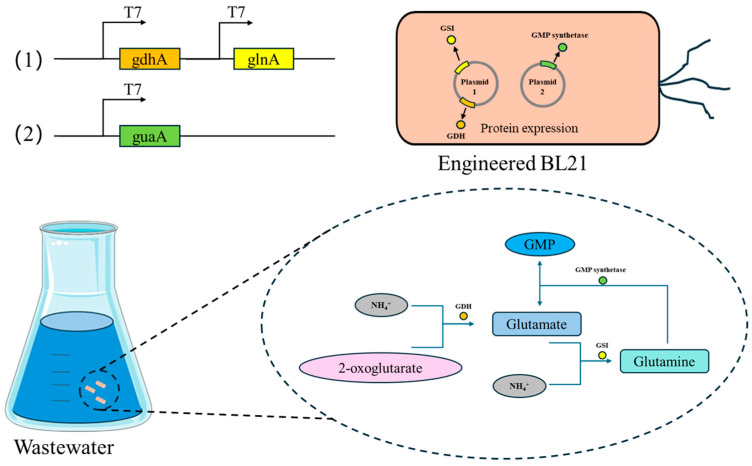
The process of constructing the engineered bacteria and the working principle of nitrogen removal. By constructing plasmids to introduce *gdhA*, *glnA* and *guaA* into *Escherichia coli* BL21, an engineering strain was constructed. The GDH pathway and GS/GOGAT pathway were used to efficiently remove nitrogen under different ammonia concentrations, and the indirect denitrification effect was achieved through the GMP synthetase encoded by *guaA* that consumes glutamine.

We therefore hypothesize that co-expressing *gdhA* and *glnA* in a fast-growing, scale-friendly chassis (*E. coli* BL21), with *guaA* positioned as a downstream nitrogen sink (a glutamine-dependent amidotransferase using glutamine—not free ammonia—as the physiological nitrogen donor) (Figure 1), will (i) enhance NH_4_^+^ assimilation under low C/N, (ii) mitigate regulatory/feedback constraints, and (iii) improve resilience to ammonium pulses. Accordingly, we (1) constructed multi-gene assimilation strains, (2) optimized expression and cultivation conditions, and (3) evaluated NH_4_^+^-N removal and shock tolerance in simulated wastewater. Throughout the study, *guaA* is treated as a downstream sink rather than a primary ammonium-assimilation enzyme. A focused synthesis of related processes (short-cut denitrification, HN-AD, and bioaugmentation/material coupling) is provided in below in Section 2 (Literature Review).

## 2. Literature Review on Biological Treatment of High-Strength Ammonia Wastewater

High-ammonia nitrogen wastewater (from livestock and poultry farming, the chemical and pharmaceutical industries, and landfill leachate) is often characterized by low C/N ratios and inhibitory substances. These conditions increase reliance on aeration and external carbon addition and result in high energy demand and operational expenditure (OPEX) for conventional nitrification–denitrification. In recent years, “low-carbon, intensive, and sustainable” biological nitrogen-removal strategies have mainly focused on four directions: (i) assimilation-oriented engineered microbial strategies, (ii) partial nitrification and its coupled short-cut processes, (iii) heterotrophic nitrification–aerobic denitrification (HN-AD), and (iv) bioaugmentation coupled with materials/processes.

Under energy and carbon constraints, short-cut denitrification (e.g., PN→nDNPR, PN→SNED and other combined processes) can simultaneously remove COD, N, and P while reducing aeration and external carbon requirements by suppressing complete nitrification, shortening the electron-acceptor pathway, and linking the metabolism of polyphosphate-accumulating organisms (PAOs) (Table S2). For example, a train of “partial nitrification + nitrite denitrification for phosphorus removal + simultaneous nitrification–endogenous denitrification” achieved deep C–N–P removal with low carbon and oxygen consumption and demonstrated cost advantages, making it suitable for complex wastewaters containing multiple nutrients [34].

HN-AD strains (such as Alcaligenes and Acinetobacter) can achieve the parallel removal of ammonia, nitrite/nitrate, and organic matter in a single reactor, and they typically exhibit rapid growth and strong tolerance to high organic loading and influent fluctuations. Using Alcaligenes faecalis WT14 as an example, its nitrogen-removal characteristics under key factors—including temperature, dissolved oxygen, pH, and C/N ratio—have been systematically characterized; related reviews also emphasize the importance of HN-AD in multi-pathway synergy and the management of free-ammonia toxicity [35].

Bioaugmentation (inoculating highly efficient functional strains/consortia, immobilization, or carrier-assisted enrichment) coupled with materials (adsorption/chemical fixation) can be superimposed on the above biological processes, substantially expanding the operational window and introducing the goal of resource recovery. Following two representative studies exemplify this approach:

(1) Iron-modified bentonite (f-MB) can efficiently and simultaneously remove phosphate and ammonium from various wastewaters, and nutrients can be recovered through regeneration, yielding slow-release fertilizers and enabling a shift from “pollution control” to “resource recovery” [36].
(2) Phoslock^TM^ used as a sediment capping material can sustainably inhibit endogenous phosphorus release even under conditions closer to real environments (anaerobic and in the presence of dissolved organic matter, DOM), underscoring the stability and practicality of the “chemical fixation + ecological restoration” coupling strategy for in-lake endogenous pollution control [37].

The assimilation pathway—via glutamate dehydrogenase (GDH) and the GS–GOGAT cycle—directly incorporates NH_4_^+^ into glutamate/glutamine, avoids gaseous NOx/N_2_ losses, and integrates nitrogen into biomass, making it an attractive nitrogen sink for low C/N systems. Prior studies have demonstrated rapid ammonium incorporation and enhanced shock tolerance in both prokaryotic and eukaryotic chassis by overexpressing nitrogen-assimilation enzymes together with transporters and carbon-flux partitioning modules. In engineered microalgae, Chlorella overexpressing cytochrome P450 has shown simultaneous removal of nitrogen and antibiotics from livestock and poultry wastewater; two-stage continuous cultivation further improves nitrogen removal and antibiotic degradation, pointing to resource-recovery-oriented wastewater treatment [38].

Compared with the above studies, the present work employs a rapidly growing, scale-friendly chassis (*E. coli* BL21) to strengthen assimilation through multi-gene synergy (*gdhA* + *glnA* + *guaA*). Approximately 90% removal was achieved at a moderate load (100 mg/L NH_4_^+^-N), and the advantage was maintained under repeated ammonium pulses, verifying the synergistic effect and shock resistance of operating GDH and GS pathways in parallel within a single host.

## 3. Materials and Methods

### 3.1. Bacterial Strains and Plasmid Constructions

The DNA primers used in this study are listed in Table S1. The *E. coli* DH5α was used for plasmid construction, and BL21 was applied for gene expression. All expression plasmids were derived from pET28a(+) or pETDuet-1. PCR-amplified DNA fragments were ligated into the linearized plasmid vectors using T4 DNA ligase (Table S1). The constructed plasmids were transformed into *E. coli* DH5α by the heat shock method. Transformants were confirmed by gene sequencing (Sangon Biotech, Shanghai, China) to verify correct inserts. Recombinant plasmids were extracted/purified using a plasmid extraction kit for subsequent experiments.

### 3.2. Culture Conditions

All *E. coli* strains were cultivated in Luria–Bertani (LB) broth (10 g/L tryptone (A300042, Sangon Biotech, Shanghai, China), 5 g/L yeast extract (A610961, Sangon Biotech, Shanghai, China), 10 g/L NaCl (Macklin, Shanghai, China)) at 37 °C with shaking at 220 rpm. The fermentation strains were grown in minimal medium (MM), which contained 1 g/L D-Glucose (A620219, Sangon Biotech, Shanghai, China), 0.5 g/L MgSO_4_·7H_2_O (Macklin, Shanghai, China), 10 g/L NaCl, 0.2 g/L KCl (Macklin, Shanghai, China), 0.24 g/L KH_2_PO_4_ (Macklin, Shanghai, China), 0.24 g/L K_2_HPO_4_ (Macklin, Shanghai, China), 0.4719 g/L (NH_4_)_2_SO_4_ (A610060, Sangon Biotech, Shanghai, China). Cultivations were conducted in open air (1 atm) using baffled 250 mL Erlenmeyer flasks (Shuniu, Sichuan, China) with a working volume of 50 mL, at 28 °C, and shaking frequency: 150 rpm. Induction at 28 °C was selected to favor soluble expression of GDH, GS, and GMPS and to limit inclusion bodies and expression burden during the 8 h induction, and this temperature was used for all screening assays. Cultures were maintained under antibiotic selection throughout seed, batch growth, and induction: kanamycin (A610029, Sangon Biotech, Shanghai, China) 50 µg mL^−1^ for pET-28a(+) Kan^R^ vectors and ampicillin (A600286, Sangon Biotech, Shanghai, China) 100 µg mL^−1^ for pETDuet-1 Amp^R^ vectors. Media were supplemented with 50 μg/mL kanamycin for pET-28a(+) constructs and 100 μg/mL ampicillin for pETDuet-1 constructs. For the two-plasmid triple-gene strain BL21(pETDuet-1-*gdhA*-*glnA* + pET-28a-*guaA*)—abbreviated in the text as BL21(pET-*gdhA*-*glnA*-*guaA*)—both antibiotics (kanamycin 50 μg/mL + ampicillin 100 μg/mL) were used during seed and induction.

### 3.3. Construction of the Recombinant Strains

The target genes (*gdhA*, *glnA*, *guaA*) were synthesized de novo (Sangon Biotech, Shanghai), cloned into pET-28a(+) or pETDuet-1, sequence-verified, and expressed in *E. coli* BL21(DE3). The Accession numbers are listed in Table 1. The cloned target fragments were ligated with the pET-28a(+) and pETDuet-1 linear empty vectors and transformed into BL21, and DNA sequencing was performed to confirm the successful cloning of the *gdhA*, *glnA*, and *guaA* genes (Table 2). The vector control is denoted BL21-EV, i.e., BL21(DE3) carrying the empty pET-28a(+) vector. The *E. coli* strains harboring the recombinant plasmids were grown in LB medium containing 100 μg/mL Kan/Amp at 37 °C on a shaking platform at 220 rpm until reaching an OD600 of 0.4–0.6. To determine optimal expression conditions, cultures were induced with isopropyl β-D-1-thiogalactopyranoside (IPTG) at final concentrations of 0, 0.1, 0.2, 0.4, 0.6, 0.8, and 1.0 mM. In addition, induction duration was varied (2, 4, 6, 8, 10, 12, or 14 h). The expression of recombinant proteins under these conditions was analyzed by SDS-PAGE and Western blot.

### 3.4. In Vitro Fermentation Test Analysis

The bacterial suspension cultured overnight was inoculated into 100 mL of sterilized simulated sewage containing the corresponding antibiotics at an inoculum volume of 10% (*v*/*v*) and cultured on a shaker at 37 °C and 220 rpm. When OD_600_ reached between 0.4 and 0.6, IPTG was added to induce gene expression, and the bacterial liquid was grown at different IPTG concentrations (0, 0.1, 0.2, 0.4, 0.6, 0.8 and 1.0 mM), different induction times (2, 4, 6, 8, 10, 12 and 14 h), and different ammonium nitrogen concentrations (100, 150, 200 mg/L) conditions, shaking at 180 rpm. The induced genetically recombinant bacteria were centrifuged to collect the bacteria, and RNA and protein were extracted for relevant detection.

### 3.5. RNA Extraction, cDNA Synthesis, and RT-PCR

Fermentation samples were washed twice with 1× PBS (E607008, Sangon Biotech, Shanghai, China), pelleted (1200 rpm, 10 min), resuspended in 800 μL TRIzol reagent, and stored at −80 °C until processing. Total bacterial RNA was extracted by TRIzol/chloroform phase separation following the manufacturer’s instructions and treated with DNase I to remove genomic DNA [39]. First-strand cDNA was synthesized from purified RNA using random hexamers. To verify transcription of the cloned genes, conventional reverse-transcription PCR (RT-PCR) was performed using gene-specific primers (sequences in Table S1), yielding amplicons of ~220 bp for *gdhA*, *glnA*, and *guaA*. The products were resolved on 1% agarose gels. No-RT and no-template controls were included to exclude genomic DNA contamination and reagent carryover [40,41].

### 3.6. Water Quality Analyses

Culture supernatants were obtained by centrifugation (8000 rpm, 5–10 min) and, where necessary, clarified by filtration. pH was measured at each sampling point using a calibrated benchtop pH meter (G001630, Sangon Biotech, Shanghai, China). NH_4_^+^-N was quantified by Nessler’s reagent spectrophotometry according to the Chinese national environmental standard HJ 535-2009 Water quality—Determination of ammonia nitrogen—Nessler’s reagent spectrophotometry (λ = 420 nm) [42]. A calibration curve was prepared with NH_4_Cl standards (specify range and R^2^), and samples were diluted to fall within the linear range. Results are reported as mg L^−1^ NH_4_^+^-N. Calibration followed the national standard (HJ 535-2009). An eight-point curve covering 0.10–2.00 mg L^−1^ yielded A_420_ = 0.1876·C − 0.0047 (C in mg L^−1^) with R^2^ = 0.9998 using a 10 mm path-length cell (Figure S3). A reagent blank was included; samples were diluted to remain within range. A mid-level check standard was run with each batch to verify calibration. Nitrate (NO_3_^−^) was determined according to the Chinese national standard HJ/T 346-2007 Water quality—Determination of nitrate-nitrogen—Ultraviolet spectrophotometry [43]. Nitrite (NO_2_^−^) was quantified with a Griess-based colorimetric kit (A038-1-1, Nanjing Jiancheng Bioengineering Institute, Nanjing, China).

### 3.7. Statistical Analysis

Data are presented as mean ± SD from *n* = 3 biological replicates unless stated otherwise. Group differences were evaluated by one-way ANOVA, followed by Duncan’s multiple range test for post hoc comparisons (α = 0.05) [44]. Assumptions of normality (Shapiro–Wilk) and homoscedasticity (Levene’s test) were examined prior to ANOVA; where assumptions were violated, Kruskal–Wallis with Dunn’s post hoc test (Holm adjustment) was applied [45]. The exact tests and significance annotations (lettering groups or asterisks) are specified in the corresponding figure legends.

## 4. Results

### 4.1. Cloning of Ammonia Assimilation Genes

**Figure 2:**
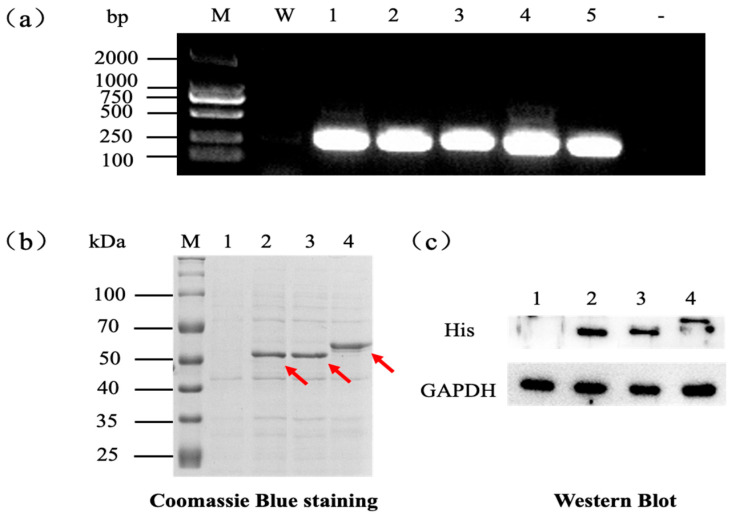
Confirmation of gene transcription and protein expression of *gdhA*, *glnA*, and *guaA* in recombinant *E. coli*. (**a**) RT-PCR analysis of target gene transcription in recombinant strains. M: DL2000 DNA marker; W: BL21-EV; 1: BL21(pET-*gdhA*); 2: BL21(pET-*glnA*); 3: BL21(pET-*guaA*); 4: BL21(pETD-*gdhA*-*glnA*); 5: BL21(pETD-*glnA*-*guaA*); –: negative control. (**b**) SDS-PAGE analysis of recombinant GDH, GS, and GMPS protein expression; red arrows indicate the target recombinant proteins. M: protein molecular weight marker; 1: BL21-EV; 2: BL21(pET-*gdhA*); 3: BL21(pET-*glnA*); 4: BL21(pET-*guaA*). (**c**) Western blot detection of His-tagged recombinant proteins. 1: BL21-EV; 2: BL21(pET-*gdhA*); 3: BL21(pET-*glnA*); 4: BL21(pET-*guaA*). GAPDH was used as the internal control.

The target genes *gdhA*, *glnA*, and *guaA* were successfully cloned into expression vectors, and their expression was confirmed in *E. coli*. PCR amplification of *gdhA*, *glnA*, and *guaA* yielded fragments of approximately 1340 bp, 1360 bp, and 1560 bp, respectively, consistent with the expected gene lengths [46,47]. Agarose gel electrophoresis (1%) confirmed that the sizes of the amplified products matched the expected lengths (Figure 2a). The PCR products were digested with two restriction endonucleases and directionally ligated into the expression vectors pET28a(+) and pETDuet-1. The recombinant plasmids were transformed into *E. coli* DH5α, and monoclonal colonies were subjected to colony PCR screening (Figure S1). Positive clones were further verified by Sanger sequencing, which confirmed the correct insertion and orientation of each target gene.

Unless otherwise stated, the empty-vector control used in this study was BL21(DE3) harboring pET-28a(+), hereafter denoted BL21-EV. For transcriptional verification, recombinant *E. coli* strains were induced, total RNA was extracted, and reverse transcription PCR (RT-PCR) was performed. Amplification of ~220 bp fragments from each transcript indicated successful transcription of the inserted genes. The confirmed plasmids were then introduced into BL21(DE3) for protein expression. Following induction with 0.2 mM IPTG at 37 °C for 6 h, SDS-PAGE analysis revealed distinct protein bands of ~50 kDa for GDH and GS, and ~58 kDa for GMPS, consistent with their calculated molecular weights when fused to the His-tag (Figure 2b,c). Western blot analysis using anti-His antibodies further validated the presence of the target proteins, confirming successful heterologous expression.

### 4.2. Optimization of Factors Affecting Protein Expression

**Figure 3:**
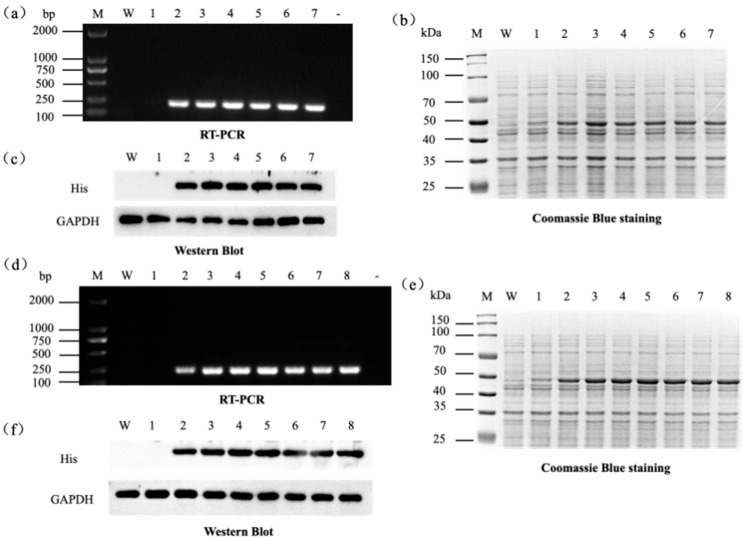
Expression analysis of the recombinant protein in BL21(pET-*gdhA*) under varying IPTG and time induction conditions. (**a**–**c**) Effects of different IPTG concentrations (0, 0.1, 0.2, 0.4, 0.6, 0.8, and 1.0 mM; lanes 1–7) on *gdhA* expression. W: BL21-EV processed in parallel under the matched condition (0.2 mM IPTG, 8 h); M: DL2000 DNA marker; –: negative control. (**a**) RT-PCR analysis of gene transcription; (**b**) SDS-PAGE analysis of recombinant protein expression, red arrows indicate the target recombinant proteins; (**c**) Western blot analysis of His-tagged GDH expression, with GAPDH as internal reference. (**d**–**f**) Effects of induction time (0, 2, 4, 6, 8, 10, 12, and 14 h; lanes 1–8) on *gdhA* expression. W: BL21-EV sampled at 14 h under identical induction; M: DL2000 DNA marker (**d**), protein marker (**e**). (**d**) RT-PCR analysis; (**e**) SDS-PAGE; (**f**) Western blot with GAPDH as internal reference.

We systematically tested different IPTG concentrations and induction times to maximize recombinant protein production. At sub-saturating IPTG levels, higher inducer concentrations led to greater mRNA and protein expression (Figure 3a–c). However, beyond 0.2 mM IPTG, we observed no further increase in protein yield. This result is substantially consistent with a previous report [48] that low IPTG concentrations (~0.05–0.1 mM) are often sufficient for maximal expression, whereas excessive IPTG can impose metabolic stress on *E. coli*, reducing cell viability and causing protein aggregation or inclusion bodies. In our study, 0.2 mM IPTG appeared to saturate expression of GDH, with higher IPTG offering no significant benefit.

We next examined the effect of induction duration on protein yield. Extending the IPTG induction period to 8 h resulted in steadily increasing GDH mRNA and protein levels (Figure 3d–f). Expression plateaued at approximately 8 h of induction, with no substantial gains thereafter. This slight decline is likely due to prolonged expression depleting nutrients, accumulating toxic byproducts, or triggering feedback inhibition in the host cells. Consistently, it has been reported that inducing cultures for more than 8 h fails to improve yields and can even harm protein quality and cell viability [49].

Based on these findings, we selected an IPTG concentration of 0.2 mM and an induction time of 8 h as the optimal conditions for all subsequent experiments, ensuring robust protein expression with minimal host cell stress.

### 4.3. Assessment of Ammonium Removal Capacity

To inform estimates of the potential for ammonium assimilation into biomass, we monitored OD_600_ every 1–2 h over 16 h under the same screening conditions (open air, baffled 250 mL flasks, 50 mL working volume, 28 °C, 150 rpm). For BL21(pET-*gdhA*-*glnA*-*guaA*), OD_600_ increased from ~0.02 at 0–1 h to ~0.61–0.64 by 8–10 h and then approached a plateau through 16 h (Figure S2). BL21-EV measured in parallel showed a comparable growth profile and plateau within the same range (Figure S2). These data indicate that biomass formation was sufficient to support assimilation-coupled ammonium removal in the subsequent assays.

**Figure 4:**
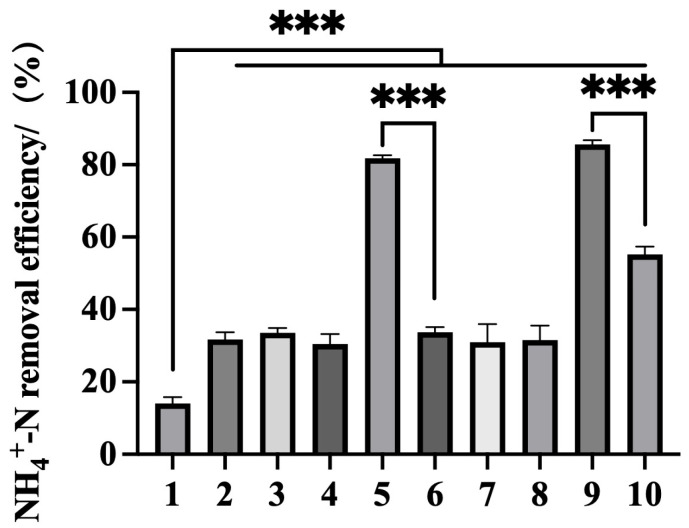
Ammonium removal efficiency of recombinant *E. coli* strains expressing different combinations of *gdhA*, *glnA*, and *guaA* genes. The bar chart represents ammonium removal rates of the following groups: 1: BL21-EV; 2: BL21(pET-*gdhA*); 3: BL21(pET-*glnA*); 4: BL21(pET-*guaA*); 5: BL21(pET-*gdhA*-*glnA*); 6: BL21(pET-*gdhA*) + BL21(pET-*glnA*); 7: BL21(pET-*glnA*-*guaA*); 8: BL21(pET-*glnA*) + BL21(pET-*guaA*); 9: BL21(pET-*gdhA*-*glnA*-*guaA*); 10: BL21(pET-*gdhA*) + BL21(pET-*glnA*) + BL21(pET-*guaA*) (*** *p* ≤ 0.001). Final broth pH remained within 7.3–7.6 (see Table S3), a range minimizing the unionized NH_3_ fraction for the tested temperature.

Using the optimized induction conditions (0.2 mM IPTG, 8 h), we evaluated the NH_4_^+^-N removal efficiency of various engineered BL21 strains expressing different combinations: two primary ammonium-assimilation nodes (*gdhA*, *glnA*) together with a downstream nitrogen-sink gene (*guaA*). In addition to strains carrying multiple genes in a single cell, we also evaluated co-cultures of single-gene strains (Figure 4). As shown in Figure 4, all recombinant strains achieved removal efficiencies ranging from 30.43% to 85.66%, all exceeding the performance of BL21-EV (14.03%). Notably, the strain co-expressing *gdhA* and *glnA* [BL21(pET-*gdhA*-*glnA*)] and the strain co-expressing all three genes [BL21(pET-*gdhA*-*glnA*-*guaA*)] were the most effective, with removal efficiencies of 81.83% and 85.66%. These results indicate that co-expressing *gdhA* and *glnA* in one host significantly enhances ammonium assimilation, consistent with the synergy of the GDH and GS branches; the additional increase observed when *guaA* is co-expressed is consistent with its role as a glutamine-consuming downstream sink rather than as a primary NH_4_^+^-incorporating enzyme.

Strains expressing only *gdhA* or only *glnA* showed moderate removal, whereas *guaA* alone had little effect, in line with its downstream positioning. Similar patterns have been reported elsewhere, where combining *gdhA* and *glnA* outperforms single-gene expression, while *guaA* alone confers only a marginal benefit [50]. Although the triple-gene strain outperformed *gdhA* + *glnA* in our assays, we treat the *guaA*-linked gain as a working hypothesis—i.e., pulling glutamine into nucleotide biosynthesis may indirectly relieve feedback/regulatory pressure on the GS/GDH branch at high flux. Mechanistic confirmation would require intracellular Glu/Gln measurements or ^15^N tracing, which were not collected here. Final broth pH at 100 mg L^−1^ NH_4_^+^-N remained in the neutral range and is summarized as mean ± SD (n) by variant: BL21-EV, 7.59 ± 0.04 (*n* = 2); BL21(pET-*gdhA*), 7.52 ± 0.03 (*n* = 2); BL21(pET-*glnA*), 7.61 ± 0.04 (*n* = 2); BL21(pET-*guaA*), 7.33 ± 0.04 (*n* = 3); and BL21(pET-*gdhA*-*glnA*-*guaA*), 7.38 ± 0.04 (*n* = 3) (Table S3). This pH band (≈7.3–7.6 at 28 °C) minimizes the unionized NH_3_ fraction, supporting an assimilation-dominated removal mechanism under our aerobic, axenic conditions.

Under the present axenic, aerobic *E. coli* conditions, NO_3_^−^-N and NO_2_^−^-N were below the method detection limits across all variants (NO_3_^−^ by HJ/T 346–2007 UV spectrophotometry with 220/275 nm correction; NO_2_^−^ by Griess kit A038-1-1 (Nanjing Jiancheng Bioengineering Institute, Nanjing, China)).

### 4.4. Removal of Ammonia Nitrogen at Varying Concentrations of Ammonium Nitrogen

Based on the ammonium removal performance observed among different recombinant BL21 strains, the triple-gene co-expressing strain BL21(pET-*gdhA*-*glnA*-*guaA*) exhibited the most effective ammonium nitrogen degradation. To further characterize its decontamination potential across ammonia loads, we conducted batch experiments using simulated wastewater with initial NH_4_^+^-N concentrations set at 100, 150, and 200 mg/L.

**Figure 5:**
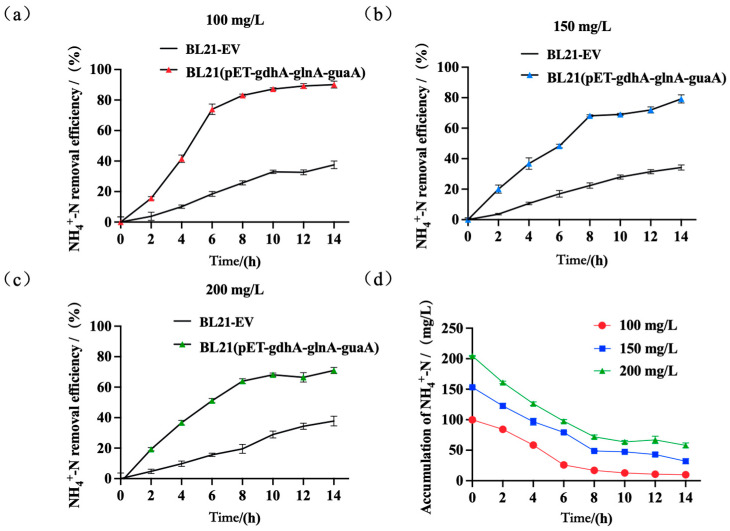
Ammonium removal by BL21(pET-*gdhA*-*glnA*-*guaA*) at varying initial NH_4_^+^-N. (**a**) Removal efficiency at 100 mg L^−1^ initial NH_4_^+^-N; (**b**) removal efficiency at 150 mg L^−1^; (**c**) removal efficiency at 200 mg L^−1^; (**d**) Final residual NH_4_^+^-N concentration (mg L^−1^) at the end of the batch assays for the three initial loads. Data are mean ± SD (*n* = 3).

The results showed that the recombinant strain maintained high removal efficiency at moderate initial concentrations but experienced some inhibition at the highest concentration (Figure 5). Specifically, at an initial 100 mg/L NH_4_^+^-N, the engineered strain achieved 90.09% removal efficiency, compared to only 37.52% removal by BL21-EV under the same conditions. When the initial ammonium was increased to 150 mg/L, the removal efficiency of the recombinant strain declined to 79.17% (still substantially higher than the BL21-EV’s 28.64%). At 200 mg/L initial NH_4_^+^-N, the recombinant’s efficiency further dropped to 70.98%, whereas the BL21-EV only reached 20.12%. Thus, as the ammonia loading increased, both strains showed reduced performance, but the triple-gene *E. coli* consistently removed a much greater fraction of ammonium than the control.

### 4.5. Efficient Ammonium Removal by Co-Expressed Recombinant Bacteria

**Figure 6:**
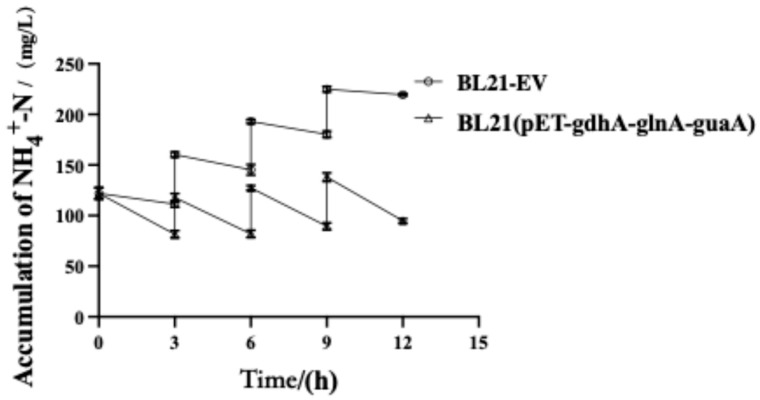
Ammonium removal performance of BL21(pET-*gdhA*-*glnA*-*guaA*) recombinant strain under repeated ammonium loading. Recombinant BL21(pET-*gdhA*-*glnA*-*guaA*) was subjected to sequential additions of 50 mg/L NH_4_^+^-N at 3 h, 6 h, and 9 h to simulate continuous ammonium input. Across the repeated 50 mg L^−1^ NH_4_^+^-N pulses, final broth pH for BL21-EV and BL21(pET-*gdhA-glnA-guaA*) remained within 7.3–7.6 (see Table S3), indicating limited NH_3_ fraction under these conditions.

In a practical wastewater treatment scenario, ammonia may enter the system in pulses or continuously increasing loads. Therefore, we evaluated the triple-gene strain’s ability to sustain ammonium removal under continuous ammonia loading. In this experiment, BL21(pET-*gdhA*-*glnA*-*guaA*) and BL21-EV cultures were subjected to three successive additions of 50 mg/L NH_4_^+^-N at 3 h, 6 h, and 9 h into the treatment period (for a total added load of 150 mg/L by 9 h). With each ammonium spike, NH_4_^+^-N concentrations were monitored to assess removal performance. As shown in Figure 6, the engineered strain consistently eliminated ammonium more rapidly than the BL21-EV control following every addition. We observed that at the 3, 6, 9, and 12 h sampling points, the engineered strain had 26.8%, 43.6%, 50.4%, and 56.8% less residual ammonium, respectively, than the wild type. Even after each ammonium spike, the recombinant maintained substantially lower NH_4_^+^-N levels.

Our findings agree with reports that engineered bacteria can adapt to repeated ammonia shocks. For instance, in one study *P. stutzeri* F2 exposed to a high ammonium load (500 mg/L) acclimated by upregulating transporter and assimilation genes, gradually restoring its removal efficiency after an initial decline [51]. Moreover, sequencing-batch and fed-batch reactors with stepwise ammonia additions often show a temporary drop in efficiency after each input, followed by recovery or a plateau as microbes adjust. In our experiments, the recombinant strain continued to outperform the wild type after each ammonium pulse, though the incremental gains diminished over time. This suggests there are limits to the strain’s compensatory response under frequent high-ammonia stress.

Overall, the continuous-loading experiment demonstrated that BL21(pET-*gdhA*-*glnA*-*guaA*) retains robust ammonia assimilation capacity even under fluctuating ammonia levels. These findings suggest that such engineered strains could be effective in dynamic wastewater treatment scenarios where ammonia input varies over time.

## 5. Discussion

In this study, BL21 strains expressing *gdhA*, *glnA*, and *guaA*—individually or in combination—were engineered to enhance NH_4_^+^-N removal from simulated wastewater. Transcriptional and translational analyses confirmed expression of all target genes. Optimal induction (0.2 mM IPTG, 8 h, 28 °C) yielded high protein levels, consistent with prior observations that moderate induction favors soluble expression and limits burden [48,52]. Under these screening conditions, growth curves recorded in parallel (Figure S2) showed comparable plateau OD_600_ between engineered strains and BL21-EV, indicating no major growth penalty within the assay window. Ammonium-removal assays further showed that co-expressing *gdhA* and *glnA* on one plasmid significantly increased NH_4_^+^-N removal, in line with their enzymatic roles: GDH incorporates NH_4_^+^ into glutamate, and GS converts glutamate to glutamine [38]. While OD_600_ traces support comparable growth under our settings, comprehensive cytotoxicity profiling (e.g., dry cell weight, viability, and cell-mass-normalized uptake, qNH_4_^+^) was not performed here and will be reported in future work.

The most effective strain was BL21(pET-*gdhA*-*glnA*-*guaA*). Because GMP synthetase (*GuaA*) is a glutamine-dependent amidotransferase that channels nitrogen from glutamine to XMP (rather than directly assimilating free NH_3_), we interpret the superior performance of the triple-gene strain as follows: the principal gain arises from the concurrent overexpression of GDH (*gdhA*) and GS (*glnA*)—the two primary ammonium-assimilation nodes—while *guaA* likely acts as a downstream nitrogen sink, drawing glutamine into nucleotide biosynthesis [53]. This pull may indirectly ease regulatory/feedback pressure on the GS/GDH branch under high assimilation flux. This sink-relief explanation is presented as a working hypothesis consistent with our data and biochemistry; we did not quantify intracellular Glu/Gln pools, GS adenylylation, or ^15^N flux into nucleotides, which will be necessary for mechanistic confirmation. Co-expressing complementary enzymes (GDH + GS) therefore augments assimilation capacity, and adding *guaA* provides an additional, context-dependent benefit by channeling assimilated nitrogen downstream [54,55].

When BL21(pET-*gdhA*-*glnA*-*guaA*) was challenged with increasing initial NH_4_^+^-N (100, 150, 200 mg L^−1^), removal efficiency declined with load. This pattern—high removal at moderate loads but loss of performance at elevated loads—agrees with prior observations (e.g., Pseudomonas stutzeri F2 under several-hundred mg L^−1^ NH_4_^+^-N) and is consistent with free-ammonia (NH_3_) toxicity and energetic/regulatory burdens on assimilation machinery at higher concentrations [51,56,57,58]. Immobilized systems often achieve near-complete removal at moderate loading (~100 mg L^−1^ within 24 h) but show reduced efficiencies and longer times at higher loads, offering an external benchmark [47]. Accordingly, selecting a suitable influent NH_4_^+^-N window will be critical for deployment. Nevertheless, even at 200 mg L^−1^, the recombinant strain substantially outperformed the control, underscoring the benefit of the engineered module under stress. Together with growth traces (Figure S2), the trends in Figure 5 and Figure 6 suggest contributions from both enhanced specific assimilation and sustained biomass; future work will report qNH_4_^+^ (per-DCW) and fit inhibition-aware kinetics.

The cooperative function and regulation of the GDH and GS pathways merit emphasis; *guaA* is considered downstream of these assimilation nodes. In BL21-EV, the GS–GOGAT and GDH routes are tightly and reciprocally controlled by nitrogen status [59,60]. Under nitrogen limitation, the NTR system upregulates *glnA* (GS) to provide high-affinity assimilation, whereas *gdhA* expression is lower; under ammonium excess, *E. coli* relies more on GDH while GS is feedback-inhibited and adenylylated. By co-expressing *glnA* and *gdhA* on the same plasmid, our strain partially bypasses these constraints, maintaining high activity in both routes. GDH provides fast assimilation of ammonia, whereas GS offers high-affinity assimilation and consumes GDH-derived glutamate to form glutamine, expanding the capacity to assimilate ammonium across a range of concentrations.

Under our axenic, aerobic *E. coli* BL21 settings, the dominant sink for ammonium is assimilation into biomass. *E. coli* lacks the dedicated ammonia- and nitrite-oxidizing machineries that define autotrophic nitrification, so formation of NO_2_^−^/NO_3_^−^ is not expected in pure-culture BL21 assays. By contrast, in non-sterile matrices or heterotrophic nitrification–aerobic denitrification (HN-AD) co-cultures, nitrite/nitrate can accumulate and should be monitored [61]. Ammonia loss to the gas phase is governed by the NH_3_/NH_4_^+^ equilibrium and gas–liquid transfer. The unionized fraction follows *f*_NH3_ = 1/(1 + 10^p*K*_a_−pH^)*f*_NH3_ = 1/(1 + 10^p*K*_a_−pH^) (p*K*_a_ ≈ 9.25 in the 25–30 °C range), so at the pH observed in our assays (≈7.3–7.6) only ~1–2% of Total Ammonia Nitrogen is present as NH_3_ at equilibrium; appreciable volatilization is typically associated with alkaline pH (≥9–10) or purpose-built stripping/recovery units. This indicates a limited volatilization sink under our shake-flask conditions, yet it warrants quantification in scale-up or higher-pH scenarios [62]. Reporting final broth pH alongside ammonium removal (Table S3) indicates that, at 100 mg L^−1^, all variants operated near neutral pH where the NH_3_ fraction is low; this is consistent with an assimilation-dominated sink rather than volatilization or oxidative pathways under the present settings.

We also note that *E. coli* can respire nitrate under anaerobic or stress conditions (DNRA/NO physiology), which is not applicable to our aerobic assays but may arise in mixed systems—underscoring the need to track NO_2_^−^/NO_3_^−^ when moving beyond axenic BL21 [63]. Overall, at 100 mg·L^−1^ NH_4_^+^-N, final broth pH was 7.33–7.61 across variants (Table S3), corresponding to ~1–2% unionized NH_3_ at 25–30 °C; this supports an assimilation-dominated sink rather than volatilization or oxidative pathways under the present axenic conditions.

Operationally, these physicochemical constraints inform where and how an assimilation module can be embedded in full-scale flowsheets. From a process standpoint, deploying recombinant assimilation strains at scale depends on siting in the flowsheet and on solids retention/removal. We envisage three scenarios. (i) Mainstream polishing in solids-retaining units (e.g., MBR, tertiary filters, IFAS): the engineered strain acts as a fast ammonium sink that dampens influent peaks; routine wasting/backwash exports assimilated N with solids, converting assimilation into net TN removal [64]. (ii) Sidestream/equalization basins with intermittent peaks (e.g., centrate/pressate): rapid NH_4_^+^ capture reduces free-ammonia shocks on nitrifiers/anammox downstream and stabilizes whole-plant performance [65]. (iii) Immobilized or encapsulated bioaugmentation, because of high local cell density, improved shock tolerance, and carrier recovery [66]. For translation, chromosome-integrated, marker-free expression and validated biocontainment (auxotrophy/kill-switch) in closed or solids-retaining units can mitigate environmental-release risk while enabling stability monitoring across SRT/HRT [67,68]. Assimilation provides rapid, growth-coupled NH_4_^+^ capture that becomes net TN removal when solids are retained/harvested; in parallel, nitrification–denitrification (or PN–anammox/PN–nDNPR) closes the nitrogen balance to N_2_ with lower aeration and carbon demand than full nitrification–denitrification, as shown by recent pilot/full-scale studies [69]. In practice, assimilation can front-load NH_4_^+^ capture (peak-shaving, low-temperature operation, co-contaminant toxicity), while PN–anammox or nitrification–denitrification downstream delivers stringent effluent TN.

Because the present work focused on assimilation-oriented engineering, NH_4_^+^-N was selected as the primary performance readout. To enable full N-balance in extended assays, in the future study, we will quantify NO_2_^−^/NO_3_^−^ (ion chromatography or standard colorimetry), total nitrogen (TN), biomass-N (OD–DCW calibration plus elemental analysis), and off-gas NH_3_ using an acid trap; Henry-law-based estimation will contextualize volatilization potential with the reported temperature and pH. These measurements are standard in wastewater nitrogen accounting and will allow closure of the nitrogen mass balance under diverse operating scenarios [3].

In summary, co-expression of *gdhA* and *glnA* on the same plasmid proved to be a key innovation for boosting *E. coli*’s ammonia assimilation, leveraging the synergy of the GDH and GS pathways. The inclusion of *guaA* provided an additional, albeit smaller and hypothesis-level, benefit by channeling assimilated nitrogen into nucleotides and potentially alleviating feedback constraints—consistent with its downstream sink role rather than a primary NH_4_^+^-incorporating enzyme.

## 6. Conclusions

In this work, we constructed a recombinant *E. coli* BL21 (pET-*gdhA*-*glnA*-*guaA*) carrying two primary ammonium-assimilation genes (*gdhA*, *glnA*) together with a downstream nitrogen-sink gene (*guaA*). We demonstrated that this engineered strain has a markedly improved ability to remove ammonium under wastewater-like conditions. The triple-gene strain achieved 90.09% removal at an initial 100 mg L^−1^ NH_4_^+^-N and maintained high performance across 100–200 mg L^−1^ as well as under repeated ammonium pulses, outperforming the empty-vector control (BL21-EV). The principal gain arises from concurrent overexpression of GDH and GS (the two assimilation nodes); GuaA contributes as a downstream glutamine sink, consistent with its role in nucleotide biosynthesis rather than direct NH_3_ assimilation.

These results highlight the potential of multi-gene co-expression strategies to improve microbial nitrogen removal. The core contribution of this study lies in proposing and validating a novel strategy of “achieving efficient NH_4_^+^-N removal through multi-gene synergistic enhancement of assimilation pathways.” However, in real wastewater systems, the effectiveness of this strategy is often severely challenged by multiple factors, including high organic loading, salinity fluctuations, and heavy metals/inhibitors. To advance this technology towards practical applications, future work will focus on the following verifiable pathways: ① integrating this enhanced assimilation pathway with existing mature COD removal and nitrogen removal processes (such as shortcut nitrification/denitrification) through parallel or series coupling to construct a comprehensive process system capable of synergistic removal of multiple pollution indicators; ② expanding microbial functional pathways in carbon source utilization, salt and toxin tolerance, and redox regulation based on modular synthetic biology strategies, while systematically evaluating their synergistic removal performance and biosafety in complex multi-pollutant scenarios. Both directions require systematic evaluation under scaled-up pilot or actual field conditions to verify the feasibility and robustness of their engineering applications.

## Figures and Tables

**Table 1:** Gene identifiers and predicted molecular weights of the enzymes expressed in this study.

Gene	Organism (Strain)	Sequence Accession (RefSeq/GenBank)	Protein Length (aa)
*gdhA*	*Enterococcus faecium*	WP_002317577.1 (RefSeq protein); genomic NZ_CP038996.1: complement(1,669,435..1,670,784); NCBI Gene ID: 66454627	449
*glnA*	*Heyndrickxia coagulans* DSM 1	WP_029141484.1 (RefSeq protein); genome NZ_CP009709.1, complement(547,556..548,893); NCBI Gene ID: 29813122	445
*guaA*	*Heyndrickxia coagulans* DSM 1	WP_029142766.1 (RefSeq protein); genome NZ_CP009709.1, complement(1,890,032..1,891,585); NCBI Gene ID: 29811950	517

**Table 2:** Recombinant *E. coli* strains constructed and their expressed genes.

Strain Name	Expression Vector	Expressed Genes	Gene Functions
BL21(pET-*gdhA*)	pET-28a(+)	*gdhA*	Encodes glutamate dehydrogenase, involved in ammonia assimilation.
BL21(pET-*glnA*)	pET-28a(+)	*glnA*	Encodes glutamine synthetase (GS), catalyzes the synthesis of glutamine from glutamate and ammonia.
BL21(pET-*guaA*)	pET-28a(+)	*guaA*	Encodes GMP synthetase (GMPS), involved in guanosine synthesis.
BL21(pET-*gdhA*-*glnA*)	pETDuet-1	*gdhA*, *glnA*	Encodes both glutamate dehydrogenase and glutamine synthetase.
BL21(pET-*glnA*-*guaA*)	pETDuet-1	*glnA*, *guaA*	Encodes both glutamine synthetase and GMP synthetase.
BL21(pET-*gdhA*-*glnA*-*guaA*) *	pETDuet1+ pET-28a(+)	*gdhA*, *glnA*, *guaA*	Encodes glutamate dehydrogenase, glutamine synthetase and GMP synthetase.

* For BL21(pET-*gdhA*-*glnA*-*guaA*), dual selection with ampicillin (100 μg/mL) and kanamycin (50 μg/mL) was applied during seed and induction steps.

## Data Availability

The data are available from the corresponding author upon reasonable request.

## Supplementary Materials

The following supporting information can be downloaded at: https://www.mdpi.com/article/10.3390/microorganisms13122646/s1, Table S1: Primers used in this study; Table S2: Qualitative comparison of ammonium assimilation vs. nitrification–denitrification and shortcut trains; Table S3: Final broth pH (mean ± SD, *n*) for the 100 mg L^−1^ NH_4_^+^-N; Figure S1: Gel electrophoresis of the *gdhA*, *glnA*, and *guaA* genes; Figure S2: Biomass growth of BL21 and BL21(pET-*gdhA*-*glnA*-*guaA*) under screening conditions; Figure S3: Calibration curve for NH_4_^+^-N by Nessler’s reagent (λ = 420 nm).

## Abbreviations

The following abbreviations are used in this manuscript:

IPTG	isopropyl β-D-1-thiogalactopyranoside
HNAD	heterotrophic nitrification–aerobic denitrification
DNRA	dissimilatory nitrate reduction to ammonium
GDH	glutamate dehydrogenase
GS	glutamine synthetase
GOGAT	glutamate synthase
COD/N	carbon/nitrogen ratio
PCR	polymerase chain reaction
LB	Luria–Bertani
Amp	Ampicillin
Kan	Kanamycin
RT-PCR	reverse transcription PCR
GAPDH	glyceraldehyde-3-phosphate dehydrogenase
HRT	hydraulic retention time
SRT	sludge retention time
