# Supplementary material for: Construction of Recombinant Escherichia coli Expressing Ammonia Assimilation Genes and Evaluation of Its Effect on Removing Ammonium Nitrogen (NH4+-N)
Source: Microorganisms. 2025 Nov 21;13(12):2646. doi: 10.3390/microorganisms13122646 (PMC12735046; doi:10.3390/microorganisms13122646)

Appendix

Table S1. Primers used in this study.

Gene Name	Forward Primer	Reverse Primer
gdhA	ATGACAAAGCAACAGAATA	ATAAGAATGCGGCCGCTCAAAC TAAACCTTCTGCTG
glnA	ATGCCTAAGTTTACACGCGA	GCAGAAGCTTTTAATACATTTGC AGGTATTGG
guaA	ATGGCAAAAGCATTCGATGT	ACAGAAGCTTCTACTCCCACTC AATCGTAG
gdhA-RNA	CGGCCCTTATAAAGGTGGAT	CAATCGATGGGCCGATATGT
glnA-RNA	CGCGACATCGTATTGGAACT	TTCACGCCAAACAACGGT
guaA-RNA	AGGATTTACGGTTGATGCCA	TTGTTCCCTACCGTTTGCC

Table S2. Qualitative comparison of ammonium assimilation vs. nitrification–denitrification and shortcut trains.

Attribute	Assimilation (this work)	Nitrification–Denitrification	Shortcut trains (PN–anammox / PN–nDNPR)
Aeration demand	Low–moderate (growth-coupled O_2)	High (full nitrification)	Low–moderate (partial nitritation/anammox reduce O_2)
External carbon demand	Low–moderate (may need modest COD for growth; sometimes endogenous COD suffices)	Often required when influent COD limited	Low (anammox no external COD; PN–nDNPR modest)
Sludge yield	Higher (N captured in biomass → must remove solids)	Moderate	Low (anammox low biomass yield)
Achievable TN endpoint	Limited unless solids are retained/removed (MBR/filters)	High (to N_2)	High (to N_2; some schemes include P removal)
Process role in plant	NH_4^+ sink/peak-shaving, polishing, low-T aid	Backbone for full TN removal	Energy/carbon-saving backbone with tighter control
Scale-up notes	Favor solids-retaining units (MBR/IFAS); consider immobilized carriers; implement marker-free integration and biocontainment	Mature, standard of care	Growing body of pilot/full-scale demonstrations; control-sensitive

Table S3. Final broth pH (mean ± SD, n) for the 100 mg L^{-1} NH_4^+–N.

Strain	pH（100 mg/L）
BL21-EV	7.59 ± 0.09
BL21(pET-*gdhA*)	7.52 ± 0.03
BL21(pET-*glnA*)	7.61 ± 0.04
BL21(pET-*guaA*)	7.33 ± 0.05
BL21(pET-*gdhA*-*glnA*-*guaA*)	7.38 ± 0.04

Figure S1. Gel electrophoresis of the *gdhA*, *glnA*, and *guaA* genes. M, DL15000 marker, (A) *gdhA* fragment; (B) *glnA* fragment; (C) *guaA* fragment.

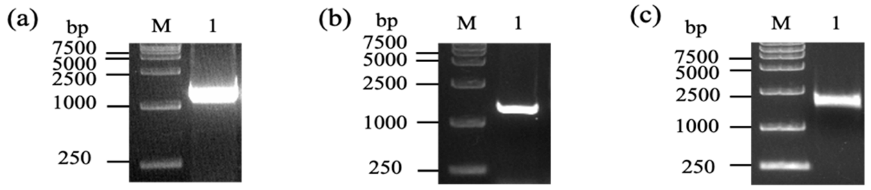

Figure S2. Biomass growth of BL21 and BL21(pET-*gdhA*-*glnA*-*guaA*) under screening conditions. (mean ± SD, n=3)

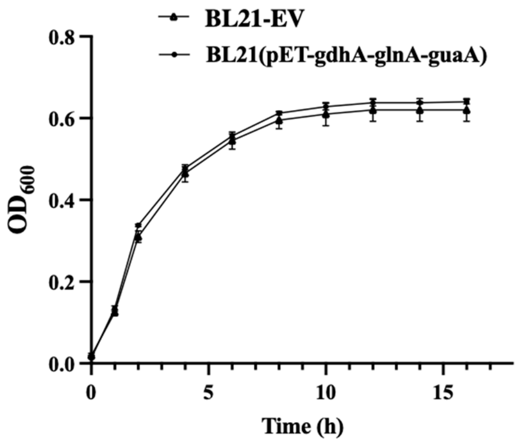

Figure S3. Calibration curve for NH_4^+–N by Nessler's reagent (λ = 420 nm). Points are mean ± SD (n = 3) for 0–2.00 mg·L^{-1} NH_4^+–N; 20-mm cuvette; 10-min color development; water blank subtracted. Linear regression: $A_{420} = 0.1876 \cdot C - 0.0047$, $R^2 = 0.9998$.

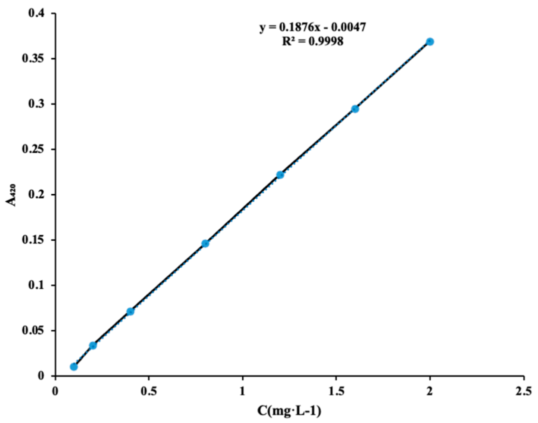

Supplement: Supplementary file 1 [file microorganisms-13-02646-s001.zip › microorganisms-3949474-supplementary.pdf]
